# Results of Surgical Treatment (Modified Sugiura-Futagawa Operation) of Portal Hypertension Associated to Complete Splenomesoportal Thrombosis and Cirrhosis

**DOI:** 10.1155/1999/30698

**Published:** 1999-04

**Authors:** Miguel Angel Mercado, Carlos Chan, Gustavo Zenteno-Guichard, Moisés Vásques, Jorge Hernández, Héctor Orozco

**Affiliations:** 1 Portal Hypertension Clinic Instituto Nacional de la Nutricion “Salvador Zubiran” México, D.F. Mexico; 2 Instituto Nacional de la Nutrición “Salvador Zubirán” Vasco de Quiroga 15 México, D.F. Tlalpan 14000 Mexico

## Abstract

*Background* Hemorrhagic portal hypertension, secondary to both intrahepatic and extrahepatic portal hypertension, is an uncommon entity. In this condition, the extrahepatic and the intrahepatic obstruction of the portal vein, due to chronic liver disease, produce a more severe form of hemorrhagic portal hypertension that is more difficult to control. The results of surgical treatment (modified Sugiura- Futagawa operation) in this subset of patients is analyzed.

*Methods* Among 714 patients with a history of hemorrhagic portal hypertension, 14 cases were found with histologically proven liver cirrhosis and complete splenomesoportal thrombosis demonstrated by means of preoperative angiography. Patients with incomplete (partial) splenomesoportal thrombosis were excluded. There were nine males and 5 females with a mean age of 51 years. Alcoholic cirrhosis was demonstrated in 50% of the cases, post hepatitic cirrhosis in 28%, primary biliary cirrhosis in 7%, and cryptogenic cirrhosis in 14%. There were nine Child-Pugh A and 5 B cases. All cases were treated by means of our modified Sugiura-Futagawa procedure.

*Results* Bleeding recurrence from esophagogastric varices was shown in one case, colonic varices in one case and hypertensive gastropathy in another of the survivors. Post operative encephalopathy was shown in 3 of the cases. The thirty-six month survival rate was 30% (Kaplan-Meier).

*Conclusions* The combination of intrahepatic plus extrahepatic portal hypertension has a worse prognosis. Treatment options are limited (sclerotherapy and/or devascularization), because shunt surgery, TIPS and liver transplantation have a very restricted role and postoperative outcome is poor.


*HPB Surgery*, 1999, Vol. 11, pp. 157-162
Reprints available directly from the publisher
Photocopying permitted by license only

© 1999 OPA (Overseas Publishers Association) N.V.
Published by license under
the Harwood Academic Publishers imprint,
part of The Gordon and Breach Publishing Group.
Printed in Malaysia.

### Results of Surgical Treatment (Modified Sugiura-Futagawa Operation) of Portal Hypertension Associated to Complete Splenomesoportal Thrombosis and Cirrhosis

MIGUEL ANGEL MERCADO *, CARLOS CHAN, GUSTAVO ZENTENO-GUICHARD, MOISÉS VÁSQUES, JORGE HERNÁNDEZ and HÉCTOR OROZCO

*Portal Hypertension Clinic, Instituto Nacional de la Nutricion, "Salvador Zubiran", México, D.F.*

*(Received 25 June 1997; In final form 12 March 1998)*

***Background*** **Hemorrhagic portal hypertension, secondary to both intrahepatic and extrahepatic portal hypertension, is an uncommon entity. In this condition, the extrahepatic and the intrahepatic obstruction of the portal vein, due to chronic liver disease, produce a more severe form of hemorrhagic portal hypertension that is more difficult to control. The results of surgical treatment (modified Sugiura-Futagawa operation) in this subset of patients is analyzed.**
***Methods*** **Among 714 patients with a history of hemorrhagic portal hypertension, 14 cases were found with histologically proven liver cirrhosis and complete splenomesoportal thrombosis demonstrated by means of preoperative angiography. Patients with incomplete (partial) splenomesoportal thrombosis were excluded. There were nine males and 5 females with a mean age of 51 years. Alcoholic cirrhosis was demonstrated in 50% of the cases, post hepatitic cirrhosis in 28%, primary biliary cirrhosis in 7%, and cryptogenic cirrhosis in 14%. There were nine Child-Pugh A and 5 B cases. All cases were treated by means of our modified Sugiura-Futagawa procedure.**
***Results*** **Bleeding recurrence from esophagogastric varices was shown in one case, colonic varices in one case and hypertensive gastropathy in another of the survivors. Post operative encephalopathy was shown in 3 of the cases. The thirty-six month survival rate was 30% (Kaplan-Meier).**
***Conclusions*** **The combination of intrahepatic plus extrahepatic portal hypertension has a worse prognosis. Treatment options are limited (sclerotherapy and/or devascularization), because shunt surgery, TIPS and liver transplantation have a very restricted role and postoperative outcome is poor.**

*Keywords:* Portal hypertension surgery, devascularization, portal vein thrombosis

### INTRODUCTION

Controversy exists between reports regarding the association of portal vein thrombosis and liver cirrhosis. Some authors state that about 10% of the patients with hepatic cirrhosis have portal vein thrombosis. Okuda *et al.*, found in an autopsy study of liver cirrhosis that the incidence of portal vein thrombosis in non splenec-

* Address for correspondence: Instituto Nacional de la Nutrición, "Salvador Zubirán", Vasco de Quiroga 15, 14000 Tlalpan, D.F., México. Tel.: (5) 573 93 21; Fax: (5) 655 10 76.

tomized patients with cirrhosis was 0.6%. Of the patients with portal vein thrombosis, about 25% were cirrhotic [1, 2].

Hemorrhagic portal hypertension in patients with liver cirrhosis and portal vein thrombosis, represents a challenge for therapeutic efforts. These patients have a mixed etiology of portal hypertension: sinusoidal component of portal hypertension due to liver cirrhosis and extrahepatic portal hypertension due to portal vein thrombosis.

Here, we report the results of the surgical treatment in patients with an uncommon etiology of portal hypertension.

### MATERIAL AND METHODS

Among 714 surgically treated patients with hemorrhagic portal hypertension in a 23 year period, the cases with histologically proven liver cirrhosis and angiographic evidence of portal vein thrombosis were selected. Portal vein thrombosis was demonstrated by angiography (celiac and mesenteric). The cases considered for this study were those in which total disappearance of the splenomesenteric and portal vein was demonstrated. Cases in which the portal vein was found with small diameter and/or intraluminal defects but still patent, were not included in the analysis.

Patients with hemorrhagic portal hypertension in the acute episode are treated by means of sclerotherapy and/or pharmacotherapy. Patients in the bleeding-free interval and with bleeding history in the last 6 months are considered for surgical treatment if they are in the low-risk group (Child-Pugh A–B cases). Patients with bad liver function (Child-Pugh C) are treated with other forms of therapy, *i.e.*, chronic sclerotherapy, B-blockers and in selected cases, intrahepatic portosystemic shunts. Some of these cases are considered for the liver transplant program. Our liver transplant program is small, doing no more than 5 cases per year. Patients with no patent portal vein are excluded from our program. Angiographic evaluation of the splanchnic area is a routine in all of our surgical patients, in order to decide on the type of surgery. Patients with adequate vessels are considered for shunt surgery (selective shunts and, in very selected cases, for low-diameter shunts). Patients with inadequate vessels are considered for our modification of the Sugiura-Futagawa operation.

In this operation an extensive devascularization of the esophagogastric area is performed. It is usually done in two stages (thoracic and abdominal). The abdominal stage is completed in the first operation. Devascularization of the stomach with ablation of left gastroepiploic vein, short gastric vessels and left coronary vein is done with interruption of the communication between the left and right gastric veins. A truncal vagotomy is done, as well as a pylorophlasty in order to prevent gastric retention. The spleen is preserved if severe hypersplenism is not demonstrated or if the patient does not have a very large spleen. In the thoracic stage, devascularization of the esophagus is done with transection in the lower third (approximately at one inch of the gastroesophageal junction). In the abdominal stage a liver biopsy is routinely obtained.

### RESULTS (TABLE I)

A total of 14 cases that met the above criteria were analyzed. Most of them were low-risk cases and liver cirrhosis was seen at macroscopic inspection while operating. Histological confirmation was also obtained in all cases. In all patients a liver biopsy was done in the abdominal stage. Liver cirrhosis from different etiologies was shown. Alcohol-related cirrhosis was demonstrated in 50% of the cases.

Preoperative splanchnic angiography showed thrombosis of the splenomesenteric and portal systems. Some of the cases had evidence of recanalized thrombosis, but an inadequate dia-

TABLE I

| | Child-Pugh | Histology | Operation | Survival (months) | Re-bleeding | Encephalo pathy | Status |
|---|---|---|---|---|---|---|---|
| F66 | B | Alcoholic cirrhosis | Abdominal stage | 32 | + | + | Lost.Variceal Bleeding |
| M61 | A | Postnecrotic cirrhosis | Thoracoabdominal | <1 | – | – | OP mortality |
| M44 | B | Alcoholic cirrhosis | Thoracoabdominal | 11 | – | + | OK grade I |
| F27 | A | Cryptogenic | Thoracoabdominal | 3 | – | – | OK |
| F65 | A | Cirrhosis | Thoracoabdominal | 48 | – | – | OK |
| M15 | B | Postnecrotic cirrhosis | Abdominal stage | < 1 | – | – | OP mortality |
| F50 | A | Postnecrotic cirrhosis | Thoracoabdominal | 72 | – | – | OK colonic bleeding |
| M74 | B | Alcoholic cirrhosis | Abdominal stage | < 1 | – | + | OP mortality |
| M65 | A | Alcoholic cirrhosis | Abdominal stage | 12 | – | – | Death myocardial infraction |
| M38 | B | Alcoholic cirrhosis | Thoracoabdominal | 2 | – | – | Death peritonitis |
| M59 | A | Alcoholic cirrhosis | Thoracoabdominal | 6 | – | – | Lost |
| M53 | A | Alcoholic cirrhosis | Thoracoabdominal | 24 | – | – | OK |
| M51 | A | Primary biliary cirrhosis | Abdominal stage | 6 | + | – | Death liver failure Hypertensive gastropathy |
| F59 | A | Postnecrotic cirrhosis | Thoracoabdominal | 36 | – | – | OK |

Nonalcoholic, negative B–C virus, negative autoinmunity.
OP = operative.

meter and blood flow were demonstrated. No vessel was found with which to perform a portosystemic shunt. This is why, all of the patients were treated by means of our modification of the Sugiura-Fatagawa operation. In 9 cases (7 Child-Pugh A, 2 B) the complete operation was done (two stages, abdominal and thoracic). In 5 cases, only the abdominal stage was done. Three of these cases died because of postoperative liver failure. The other two cases were lost to follow-up and the operation could not be completed.

From the 9 survivors with the complete procedure, the following data were obtained.

### Re-bleeding

Three of the patients had re-bleeding, one because of esophagocardial varices one because of colonic varices and the other because of hypertensive gastropathy. Thus, global bleeding recurrence was 33%, but from esophagogastric varices it was 11%.

### Encephalopathy

This was assessed by means of clinical evaluation (including number connection tests) and was demonstrated in 3 cases; grade II – III in two cases and grade I in one case. Excluding patients with operative mortality and those lost for follow-up, the encephalopathy frequency was 33%.

### Survival

This was calculated by means of the Kaplan-Meier method. Survival was 30% at 36 months. Table I shows the individualized outcome of the patients.

### DISCUSSION

Hemorrhagic portal hypertension, with an intra and extrahepatic component, is a rare condition. It represents only 2% of the cases operated in our hospital. According to Okuda *et al.*, portal vein thrombosis in cirrhotic patients is also a rare condition with a very low frequency in the Japanese population. Although some interesting explanations of the etiology have been given, the exact physiopathology is unknown. Certainly the flow alterations in the portal vein secondary to hypertension and possibly the underlying liver disease may play a role [3]. Also the kind of thrombus and its evolution may differs from one patient to another [4].

To our knowledge, the treatment results with devascularization procedures in this subset of patients have not been previously reported.

The combination of liver cirrhosis and splenomesoportal system thrombosis represents 2% of cases from our surgically treated population. These had splenic mesenteric and portal veins unsuitable for shunting, with chronic obstruction. The veins were tortuous, with a critical reduction of their diameter, showing a small lumen and not appropriate for any kind of shunt (total, selective or low diameter). Under these circumstances, to perform a shunt is not advisable. For these cases, we believe devascularization procedures (modified Sugiura-Futagawa operation) do not have good long term results.

In this series, the frequency of recurrent bleeding was 33% as well as the frequency of encephalopathy, the survival rate was low. These results are different when compared to those obtained in low risk patients (Child A – B) in which usually a low rebleeding rate (10%) and postoperative encephalopathy (4 – 7%), as well as a good long term survival is obtained [5 – 9]. In the cirrothic-thrombotic patient a higher operative mortality has been reported, we think that this is the result of a more complex operation with a higher degree of technical difficulty. The devascularization is difficult due to the collateral circulation and higher intraoperative bleeding with an increased requirement for intraoperative blood products. In our experience, this fact together with a long operative time, promotes a poor postoperative outcome; it has also been our experience that these cases more frequently develop liver failure and multiorgan failure as well as postoperative coagulopathy.

It may be that the mixed etiology, (intra and extra hepatic) of the portal hypertension plays a role in the poor outcome. For patients with a non cirrhotic liver and extrahepatic portal hypertension, collaterals, have an hepatopetal flow tendency, because the liver represents a low-resistance vascular area. In the cases analyzed in this paper, the liver represents a high resistance area, and this is why more portosystemic collaterals are promoted, explaining the high encephalopathy rate and the neoformation of esophageal and gastric varices after the devascularization. This translates in to a higher rebleeding rate.

These cases represent a challenge for the treatment of hemorrhagic portal hypertension and other therapy choices have also a limited role. Pharmacotherapy for secondary prophylaxis has a high rebleeding rate in all subsets of patients with portal hypertension. Transendoscopic sclerotherapy and/or banding is one of the choices, but a high rebleeding rate is to be expected [11]. No data is available in the literature in the cirrhotic-thrombosed subset of patients.

A shunt can not be done safely in these cases, because an adequate vessels can not be found to perform either a total, selective or low diameter shunt; in selected cases a makeshift shunt can be performed with one dilated vein found in the splanchnic area [12], but they have a high dysfunction rate and obstruction can be expected in most of them.

Liver transplantation represents a challenge for the surgeon in these patients. Nevertheless, there are cases in which a thrombectomy of the portal vein and/or a bypass of the recipient mesenteric vein to the donor portal vein is achievable [13].

Although some successful cases with portal vein thrombosis treated by means of transjugular shunts have been reported, it's role in this subset of patients has not been established.

In conclusion, bleeding portal hypertension of mixed etiology (intra and extrahepatic) is a rare condition and devascularization procedures don't offer a good long term result.

### References

[1] Jones, C. A. (1965). In: Bockus, H. L. (Ed.). *Diseases of the hepatic blood vessels*, Vol. 3, 2nd de. Philadelphia: W. B. Saunders, 441–6.

[2] Okuda, K., Ohnishi, K., Kimura, K., Matsutani, S., Sumida, S., Goto, N., Mucha, H. *et al.* (1985). Incidence of portal vein thrombosis in liver cirrhosis. An angiographic study in 708 patients, *Gastroenterology*, **89**, 279–286.

[3] Wanless, IR, Wong, F., Bledins, L. M., Greig, P., Heathcote, E. J. and Levy, G. (1995). Hepatic and portal vein thrombosis in cirrhosis: possible role in development of parenchymal extinction and portal hypertension, *Hepatology*, **21**(5), 1238–1247.

[4] Orloff, M. J., Orloff, M. S., Orloff, S. L. and Girard, B. (1997). Portal vein thrombosis in cirrhosis with variceal hemorrhage. *J. Gastrointestinal Surg.*, **1**, 123–131.

[5] Orozco, H., Mercado, M. A., Takahashi, T., Hernández, J., Capellán, J. F. and García-Tasao, G. (1992). Elective treatment of bleeding varices with the Sugiura operation over 10 years, *Am. J. Surg.*, **163**, 585–589.

[6] Orozco, H., Takahashi, T., Mercado, M. A., Prado, E. and Chan, C. (1994). Surgical management of extrahepatic portal hypertension and variceal bleeding, *World J. Surg.*, **18**, 246–250.

[7] Orozco, H., Mercado, M. A., Takahashi, T., Rojas, G., Hernández, J. and Tielve, M. (1994). Survival and quality of life after portal blood flow preserving procedures in patients with portal hypertension and liver cirrhosis, *Am. J. Surg.*, **168**, 10–15.

[8] Warren, W. D., Milikan, W. J., Smith, R. B., Rpins, E. B., Henderson, J. M., Salam, A. A., Hersh, J. *et al.* (1980). Noncirrhotic portal vein thrombosis: Physiology before and after shunts, *Ann. Surg.*, **192**, 341–349.

[9] Orozco, H., Takahashi, T., Mercado, M. A., García-Taso, G. and Hernómdez, J. (1991). The Sugiura procedure for patients with hemorrhagic portal hypertension secondary to extrahepatic portal vein thrombosis, *Surg. Gynecol. Obst.*, **173**, 45–48.

[10] D'Amico, G., Pagliaro, L. and Bosch, J. (1995). The treatment of portal hypertension. A Meta-Analytic Review, *Hepatology*, **22**, 332–354.

[11] Henderson, J. M., Kutner, M. L. T., Milikan, W. J., Galambos, J. T., Riepe, S. P., Brooks, W. S., Bryan, C. and Warren, W. D. (1990). Endoscopic variceal sclerosis compared with distal splenorenal shunt to prevent current variceal bleeding in cirrhosis. A prospective randomized trial, *Ann. Intern. Med.*, **112**, 262–269.

[12] Warren, W. D., Milikan, W. J., Henderson, J. M., Rasheed, M. E., Salam, A. *et al.* (1984). Selective variceal decompression after splenectomy or splenic vein thrombosis with a note on splenopancreatic disconnection, *Ann. Surg.*, **199**, 694–702.

[13] Langnas, A. N., Marujo, W. C., Stratta, R. J., Eood, P., Ranjan, D., Ozaki, C. and Shaw, B. W. (1992). A selective approach to preexisting portal vein thrombosis in patients undergoing liver transplantation, *Am. J. Surg.*, **163**, 132-136.
[14] Radosevich, P. M., Ring, E. J., La Berge, J. M., Pletzer, M. Y., Haskal, Z. J., Doherty, M. M. and Gordon, R. L. (1993). Transjugular intrahepatic protosystemic shunts in patients with portal vein occlusion, *Radiology*, **186**, 523-527.

### COMMENTARY

The issue of treating patients affected by cirrhosis, upper G. I. bleeding and splenomesoportal thrombosis still remains an unsolved problem due to the severity of combined pre and intrahepatic portal hypertension. The nice Mercado experience showed how this modified Sugiura-Futagawa operation in patients with relatively good hepatic function (Child A-B) was partially unsuccessfull due to the high perioperative and medium term mortality. However, the lack of a randomized clinical trial comparing this therapeutric approach to the "natural history" of these patients, makes every comment unopportune. It is probably a certainity that the role of T.I.P.S.S. as well that of shunt surgery is negligible.

On the contrary, we believe that the field of transplantation will possibly find an acceptable terapeutic solution for these patients. Recently, Andreas Tzakis described a new technique for liver transplantation in presence of complete splenomesoportal thrombosis using a "caval transposition". In short, the venous inflow to the graft is obtained by a cavo-portal anastomosis performed after interruption of the IVC above the renal veins. This technique may not improve the portal hypertension; oppositely, after the operation the patient may experience a worsening of portal hypertension due to the increased pressures in the IVC. However, after the transplant these patients shift from a severe pre and intrahepatic portal hypertension with impaired hepatic function, to a severe prehepatic hypertension associated with normal intraportal pressures and improved liver function. Therefore, we can theoretically conclude that in these patients the association of a Sugiura-Futagawa procedure (or other deconnection procedures) to liver transplantation might be better tolerated thanks to the basically normal function of the new liver. Such an association might be excuted in one or more steps depending on the clinical conditions of the patients at the time of transplantion. At present, of course, some more studies are needed.

Prof. D. V. D'Amico
Via San Marco 9
35030 Selvazzano
Padova
Italy

Mercado *et al.*, describe their experience with a difficult topic, *i.e.*, the surgical treatment of portal hypertension in cirrhotic patients with complete splenomesoportal thrombosis. In such a setting, the therapy used by the authors carries a high mortality rate and also a high morbidity rate. However, experience in the literature remains limited due to the rarity of these patients.

Through their experience, two main points deserve comments.

First, what is the pathophysiology of portal hypertension in these patients? The answer to this question should provide on explanation to understand why in a subgroup of patients surgery carries a good prognosis. conversely, this answer should also allow selection of other therapies for patients carrying a poor prognosis.

Second, the absence of trials including variceal banding in such patients must be addressed in view of the data provided by Mercado *et al.*

Prof. Y. Horsmans
Universite Catholique de Louvain
Cliniques Universitaire Saint -Luc
Avenue Hippocrate 10
20 Bruxelles
Belgium

